# Origins of the Domestic Dog and the Rich Potential for Gene Mapping

**DOI:** 10.4061/2011/579308

**Published:** 2011-01-17

**Authors:** Jeremy R. Shearman, Alan N. Wilton

**Affiliations:** ^1^School of Biotechnology and Biomolecular Sciences, University of New South Wales, Sydney, NSW 2052, Australia; ^2^Clive and Vera Ramaciotti Centre for Gene Function Analysis, University of New South Wales, Sydney, NSW 2052, Australia; ^3^National Center for Genetic Engineering and Biotechnology, 113 Phahonyothin Road Klong 1, Klong Luang, Pathumthani 12120, Thailand

## Abstract

The unique breeding structure of the domestic dog makes canine genetics a useful tool to further the understanding of inherited diseases and gene function. Answers to the questions of when and where the dog was domesticated from the wolf are uncertain, but how the modern diversity of dog breeds was developed is documented. Breed development has resulted in many genetically isolated populations which are segregating for different alleles for disease and morphological and behavioral traits. Many genetic tools are available for dog research allowing investigation into the genetic basis of these phenotypes. Research into causes of diseases in dogs is relevant to humans and other species; comparative genomics is being used to transfer genetic information to them, including some studies on morphological and behavioral phenotypes. Because of the unique breed structure and well-maintained pedigrees, dogs represent a model organism containing a wealth of genetic information.

## 1. Domestication from the Wolf

Domestic dogs can be viewed as one of mankind's largest and longest running breeding experiments. The process has resulted in over 400 breeds with considerable morphologic and behavioral diversity compared to the gray wolf ancestor. The origin and time frame of domestication from the gray wolf are hotly debated. Early work using phylogenetic substitution rates in mitochondrial D-loop sequence suggests that dogs might have originated as early as 100,000 years ago [1, 2]; however, this figure is based on an unlikely assumption of a single founding mtDNA haplotype. A similar study by Savolainen et al. [3] using samples from a more widely distributed area and allowing for multiple mtDNA haplotypes in the founding population suggested a domestication time of 15,000 years ago. Pang et al. [4] using entire mitochondrial genomes from 169 dogs and mitochondria control region sequence data from 1543 dogs suggest a domestication time of 5,400 to 16,300 years ago. However, dog-like fossils have been dated as early as 31,000 years ago [5]. The discrepancy between genetic and archaeological data could be caused by several things. One is incomplete separation of wolf and dog populations with recent admixture, as has been observed in a US wolf population, which would reduce the apparent time since domestication [6].

Identifying the location of dog domestication has proven difficult, partly because it is confounded by the choice of samples from wild relatives for comparison to dog. Mitochondrial DNA (mtDNA) data has been used to support East Asia as the origin of all modern dog breeds [3, 4]. Verginelli et al. [7] have used mtDNA to suggest an Eastern European origin of domestication. The number of dogs included from a region's native dog population can influence the conclusions as shown by Boyko et al. [8], who examined mtDNA sequence from native African village dogs (representing domestic dogs prior to breed development). They suggested that genome-wide autosomal markers were required to answer the question of where dogs were first domesticated. vonHoldt et al. [9] typed a set of 48,000 SNPs on Affymetrix mapping array version 2 in 912 dogs from 85 modern and ancient breeds and 225 gray wolves and concluded that dogs were likely domesticated from multiple locations. Some ancient breeds seem to have a primary ancestry in East Asia, but the majority of breeds have ancestry in the Middle East [9]. There were no clines of genetic diversity in dog populations, so unlike in human populations, in dog populations, genetic diversity cannot be used to trace ancestral origins of dog. The data leads one to speculate that there were multiple origins of domestication, but this does not fit well with the global distribution of all mtDNA clades [4].

## 2. Dog Population Structure

The domestication of the wolf established populations of native dogs in several places around the world, and these native populations existed for some time allowing some genetic diversity to rebuild after the original domestication bottleneck. The breed structure and relationship between dog breeds can be teased apart using SNP data such as that from vonHoldt et al. [9]. The ancient dog breeds, such as the Australian Dingo, Basenji, and Chinese Shar Pei were isolated from these early dogs thousands of years ago and remained more or less a distinct breeding population [9, 10]. Most modern dog breeds have been developed in the last two hundred years [11, 12] by selecting dogs with certain phenotypes, primarily in Europe [9]. Line breeding (inbreeding) with strong artificial selection for generations has resulted in different characteristics becoming fixed in each breed. Today, dogs have one of the most diverse phenotypic ranges of any species [13]. Dog breeds can be classified into nine groups based on form or function: toy dogs, spaniels, scent hounds, working dogs, mastiff-like breeds, small terriers, retrievers, herding breeds, and sight hounds [9]. Dogs within a classification tend to be more similar genetically and grouped together in neighbour-joining trees performed on SNP data [9]. Breeds of dog generated by crossing dogs from two different groups are also reflected in the neighbour-joining tree as having ancestry to both groups [9]. Understanding the origins of a breed is important for genetic studies as the history can give an indication of potential allele sharing between breeds.

For a dog to be classified as purebred, it has to be the offspring of purebred parents. Pedigree records are well documented, and dogs of mixed ancestry are excluded from any breed. This unique population structure results in a significant degree of inbreeding and strong population substructure. One factor that influences these processes is the popular sire effect. A popular sire is a male that is highly sought after for breeding purposes, usually from winning dog shows or herding competitions. A popular sire can produce hundreds of offspring, contributing significantly to the gene pool of the next generation [14]. This results in inbreeding effective population sizes that are around 50 individuals for each breed [14]. Such purebred populations have strong genetic drift which can result in genetic diseases or inbreeding depression in the breed. A dog carrying a recessive disease allele can pass it on to hundreds of offspring rapidly spreading it through the population. Most purebred dogs will carry several such disease alleles, and many of them will be unique to the breed due to new mutations or drift increasing the frequency of a mutation present in a founder dog.

Breeding of dogs is easily manipulated and planned. Single animals with a rare disorder can be bred into disease colonies for research. Crosses between breeds can be used to place genes for genetic traits in different genetic backgrounds to allow the study of the influence of modifying genes on phenotype. This can be important when studying diseases with low penetrance or variable expression.

## 3. Gene Mapping in Dogs

Dogs have large haplotype blocks (regions of linked alleles in strong linkage disequilibrium; see [15] for review of linkage disequilibrium and haplotype blocks) within a breed and smaller haplotype blocks between breeds. A haplotype block is a long stretch of DNA with a particular combination of allelic variants that often occur together, and in canines these haplotype blocks can be up to ten times the length of haplotype blocks found in humans [12, 16]. Large haplotype blocks allow mapping in dogs to be performed with fewer polymorphic markers and fewer individuals as compared to human studies and make purebred dogs an ideal model for the study of genetic traits and diseases. However, large haplotype blocks also mean that any trait region identified within a single breed can be in the range of several Mb incorporating tens of genes to over a hundred [10, 17]. Such a large trait region is a significant problem for identifying causative mutations. In some cases this problem can be overcome by using related breeds that share the same trait to help narrow the interval containing the mutation [18]. For cases where the trait of interest is restricted to a single breed, a search may indicate a large possible trait region requiring a candidate gene approach to be applied within that region. 

For identifying novel genes involved in genetic pathways, the study of canine traits can be useful. Conditions that are rare in outbred populations, such as human, can become common within one or several inbred breeds, and so there are many traits that can be readily studied in dogs. Dogs typically have less heterogeneity (multiple alleles causing indistinguishable phenotypes) than an outbred population which means that analysis of a canine phenotype will result in a stronger genetic signal. Mapping canine homologues of complex traits is therefore likely to identify single, high-effect loci as a result of the breeding structure [19]. Dog genetics may not hold all the answers to the causes of complex trait phenotypes in outbred populations, but it can shed light on at least some genes and pathways involved. Dogs can make a good model organism as they generally share the same environment as humans, supplementing the use of mouse, zebra fish, and yeast as models.

A typical mapping experiment in dogs would make use of an association study using SNP arrays on a trait or set of traits that exists in multiple breeds such as coat variation [19]. In the example of coat variation, three phenotypes were each mapped within a single breed: obvious moustache and eyebrows, hair length, and curled hair. Mapping was then expanded to include dogs from 80 breeds allowing the authors to exclude false positives caused by sample stratification and to narrow the candidate region by taking advantage of the smaller haplotype block sharing between breeds. This made the identification of genes simpler.

## 4. Tools Available

A 1.5x coverage sequence of a poodle [20] and a 7.5x coverage sequence of a boxer [12] have provided an annotated dog genome and allowed for comparative genomics and the establishment of the dog as a mammalian model organism. Other important milestones were the development of a canine expression array (Affymetrix) and several canine SNP arrays with 100,000s of loci ([18], http://www.affymetrix.com/estore/, http://www.illumina.com/). SNP arrays replace the need to laboriously type large numbers of microsatellites for whole-genome analysis. A comprehensive linkage map for all dog chromosomes is now also available that can be used in conjunction with whole-genome mapping [21]. The availability of high-throughput genotyping technologies allows for large-scale mapping experiments to be rapidly performed with markers spaced densely enough that fine mapping to localize the gene after initial mapping studies will be easier or may even be unnecessary.

With the development of next generation sequencing, which allows gigabases of DNA sequence to be generated from a single sample (see [22] for review of next generation sequencing), several technologies have become available to address the issue of targeting particular part(s) of the genome to be sequenced [23–25]. Sequence capture involves using many DNA probes giving sequence representation of a target sequence to hybridise with DNA from the sample and temporarily capture specific target regions of the genome that are then recovered. Sequence capture followed by next generation sequencing is useful when a trait or disease gene is mapped to a region of several megabases in size [18].

## 5. Disease Genetics

The gene complement of most eutherian mammals is very similar, and dogs have similar genetic diseases to those observed in large outbred populations, such as humans, from simple monogenic traits to complex disorders. The medical attention we provide our much loved canine companion has led to an extensive list of known disorders, second only to human and mouse (see [26]: Inherited Diseases In Dogs database, http://server.vet.cam.ac.uk/index.html). Information on the genetic basis of common complex disorders such as cancers, heart diseases, and diabetes in the dog can be informative for disease gene identification in other species. A benefit of using the dog model for disease studies is the well-documented pedigrees providing information on relatedness, inbreeding coefficients, common ancestors, and thus high-risk family lines. This information can aid in the selection of samples for genetic studies on diseases and traits by allowing the researcher to identify potential carriers of a disorder.

Cancers of many different types exist in different breeds offering the potential for insight into disease mechanisms and treatment options for cancers in humans and other species and this is a major research focus of several groups, for example, LUPA (http://www.eurolupa.org/). For example, there is a familial medullary thyroid cancer common in the Alaskan Malamute [27], a Non-Hodgkin's lymphoma common in the Boxer, Setter and Cocker Spaniel [28], and mammary tumours in the English Springer Spaniel [29] to name a few. The types of cancers observed in dogs are, in many cases, similar to forms found in humans. Gene expression profiling of 32 cases of canine osteosarcoma has identified expression patterns associated with short- versus long-term survival similar to those found in humans [30]. Genomic regions with copy number abnormalities that were identified in cases of canine colorectal cancer contain many genes known to be disrupted in human colorectal cancer. Furthermore, clustering of human and dog copy number abnormalities grouped samples into tumour subtypes rather than species [31]. The genetic similarities in cancer subtypes between human and dog suggest that genetic pathways leading to cancer may be similar across species. Cases of canine hemangiosarcoma have also been suggested as good models to study the effect of cancers in varying genetic backgrounds, because the genetic stratification amoung dog breeds is somewhat similar to the genetic stratification observed among different human ethnicities [32, 33]. 

Dogs also suffer from inherited high blood pressure and various cardiovascular disorders such as arrhythmias, cardiomyopathy, and dilated cardiomyopathy. Dilated cardiomyopathy in dogs presents with clinical signs similar to human symptoms such as shortness of breath, decreased appetite, weakness, and collapse. Interestingly, individual breeds differ in which of these clinical signs is the most common [34]. Dogs suffer from many immune-mediated disorders, similar to humans (see [35] for review of immune disorders), which may be due to disease alleles at several loci segregating within dog populations, a shared environment with humans or a combination of both of these factors. These examples represent naturally occurring diseases of biomedical significance, segregating in purebred dog populations. Mapping of these disease genes in the dog could aid in elucidating the disease mechanism in humans and other species. The above examples are areas where canine genetics could significantly aid the understanding of complex disease. 

Two examples where canine genetics has shed light on previously unknown disease mechanisms in humans include the discovery of a narcolepsy gene in dogs, *HCRTR2* [36, 37] and a novel photoreceptor gene, *PRCD*, involved in cases of retinitis pigmentosa [38]. Other cases where mapped dog diseases have been speculated as corresponding to unmapped homologous diseases in human include a duplication of four genes predisposing to dermal sinus, which in humans is often associated with spina bifida [39] and a set of five loci that are associated with systemic lupus erythematosus [40]. In most cases the identification of the cause of a canine disease identifies a gene where mutations in homologs cause a similarly characterised disease in other species. Identifying canine homologues to human disease genes allows the usage of affected dogs as a mammalian model to further study the disease mechanism and potential treatment options.

## 6. Morphology and Behavioural Genetics

There is also potential from canine genetics to identify the genetic basis for morphological and behavioural traits. Any dog chosen from a purebred population will be morphologically defined by the breed-defining traits, and thus measurements of characters are not required for all individuals when comparing across breeds [41]. Consistent phenotypes mean that SNP data from multiple studies can be pooled and used to map genes for these breed-defining traits. Examples where large datasets incorporating large numbers of breeds have been used to map such traits are beginning to appear such as the coat variation study by Cadieu et al. [19]. 

Loss-of-function mutations in myostatin (*MSTN*) is a good example of a trait transferrable between species using comparative genomics. It causes increased muscle mass in several species, including dogs and horses (see [42] for review). Heterozygosity for a *MSTN* mutation has been found to increase racing ability in both whippets and racing horses [43, 44]. Whippets are a racing dog breed that have been selected for a combination of slim build, deep chest, and powerful legs allowing them to reach speeds over 50 km per hour. Analysis of the genetic factors behind the whippet phenotype and running speed may complement studies into the genetics of running speed in thoroughbred racehorses. Understanding the genetics of traits such as skeletal structure, muscle density, and muscle mass would benefit breeding studies on these species and others. 

One of the most remarkable characteristics of domestic dogs is their ability to pick up and understand human cues and emotions. Dogs show a strong attachment relationship with their caregiver and are more amenable to training than wolves raised in the same environment [45–47]. This suggests that the characteristics that allowed dogs to be domesticated have a genetic component. vonHoldt et al. [9] have found a strong selection signal in domestic dogs on a gene, *WBSCR17*, which in humans is involved in William-Beuren syndrome, a disease that includes mental retardation, ease with strangers, and a desire to be in groups. Such characteristics would make dogs easier to handle and could have been strongly selected early during the domestication process.

Individual dog breeds are enriched or fixed for innate behavioural characteristics including pointing, herding, and aggressive behaviour. While these breeds still require training for pointing and herding, they are far more responsive to the training than other breeds, which suggests that these traits have a degree of genetic predisposition. Mapping for these traits has identified genomic regions that appear strongly associated with these behaviours [48]. Different dog breeds show variation in the amount of confrontational or aggressive behaviour they exhibit towards humans and other dogs. Takeuchi et al. [49] have mapped a trait they call “aggression towards strangers” to a variant in *SLC1A2*, which may be responsible for overly aggressive behaviour. These few examples show how canine genetics can be used to identify genes potentially affecting behaviours, which may assist in identifying similar genes affecting behaviour in other species.

## 7. Conclusion

Dogs represent such a rich potential resource to further the understanding of diseases and genetic traits because of their history of domestication and breed development. Domestication of the dog has resulted in many isolated populations, much like a breeding experiment with gene mapping as the aim. Recent advances in understanding this genetic history are important for mapping genes for various phenotypes and traits. Breeds fixed or highly enriched for certain phenotypes already exist. Identifying the genetics responsible for breed-defining phenotypes is potentially as simple as collating the existing SNP array data and performing the analyses. A confounding issue that could pose a problem for mapping the phenotypes listed above is sample stratification and the large haplotype blocks that exists within breeds. However, canine genetics has significant potential to contribute to the understanding of genetic disorders and functional genomics in other species and will compete with other species as a genetic model organism.
